# Root Carbon Resources Determine Survival and Growth of Young Trees Under Long Drought in Combination With Fertilization

**DOI:** 10.3389/fpls.2022.929855

**Published:** 2022-06-03

**Authors:** Yue Yang, Shengnan Ouyang, Arthur Gessler, Xiaoyu Wang, Risu Na, Hong S. He, Zhengfang Wu, Mai-He Li

**Affiliations:** ^1^College of Ecology and Environment, Hainan University, Haikou, China; ^2^Swiss Federal Institute for Forest, Snow and Landscape Research WSL, Birmensdorf, Switzerland; ^3^Key Laboratory of Geographical Processes and Ecological Security in Changbai Mountains, Ministry of Education, School of Geographical Sciences, Northeast Normal University, Changchun, China; ^4^Institute for Forest Resources and Environment Research Center of Guizhou Province, Guizhou University, Guiyang, China; ^5^Institute of Terrestrial Ecosystems, ETH Zurich, Zurich, Switzerland; ^6^Jiyang College of Zhejiang A and F University, Zhuji, China; ^7^School of Geographical Sciences, Inner Mongolia Normal University, Hohhot, China; ^8^School of Natural Resources, University of Missouri, Columbia, MO, United States

**Keywords:** non-structural carbohydrates, NSC, over-winter carbon consumption, photosynthesis, recovery, water deficit

## Abstract

Current increases in not only the intensity and frequency but also the duration of drought events could affect the growth, physiology, and mortality of trees. We experimentally studied the effects of drought duration in combination with fertilization on leaf water potential, gas exchange, growth, tissue levels of non-structural carbohydrates (NSCs), tissue NSC consumption over-winter, and recovery after drought release in oak (*Quercus petraea*) and beech (*Fagus sylvatica*) saplings. Long drought duration (>1 month) decreased leaf water potential, photosynthesis, and NSC concentrations in both oak and beech saplings. Nitrogen fertilization did not mitigate the negative drought effects on both species. The photosynthesis and relative height increment recovered in the following rewetting year. Height growth in the rewetting year was significantly positively correlated with both pre- and post-winter root NSC levels. Root carbon reserve is critical for tree growth and survival under long-lasting drought. Our results indicate that beech is more sensitive to drought and fertilization than oak. The present study, in a physiological perspective, experimentally confirmed the view that the European beech, compared to oak, may be more strongly affected by future environmental changes.

## Introduction

Global climate change has led to and is continuously resulting in increases not only in drought intensity but also in the frequency and duration of drought events globally (IPCC, 2013). Two main hypotheses have been proposed and are currently debated to explain the mechanisms for the widespread forest dieback caused by increased drought events (Hartmann et al., 2018; Spinoni et al., 2018): trees would die (1) due to hydraulic failure or (2) as a result of carbon starvation (McDowell and Allen, 2015; Gessler et al., 2018). The hydraulic failure hypothesis proposes that the tree mortality results from the embolism of the xylem vessels under high evaporative demand and restricted soil water availability (Nardini et al., 2013), whereas the carbon starvation hypothesis suggests that tree mortality would be caused by a carbon supply limitation due to stomatal closure and thus reduced photosynthesis that cannot cover the carbon and energy demand for maintenance processes (McDowell et al., 2008; Rowland et al., 2015). Concerning carbon starvation, several studies have shown that the contents of starch, a compound that serves as carbon storage and is build up when assimilation exceeds plant’s demand for carbon, were strongly reduced by severe soil water deficit (McDowell, 2011), implying carbon limitation (Reinhardt et al., 2015). Previous studies also suggested that hydraulic perturbation could prevent phloem transport (Sevanto, 2014) and consequently constrains carbon accessibility (Sala et al., 2012; Hartmann and Trumbore, 2016), even though the carbon availability in the tree crown area might not be restricted. Thus, hydraulic failure and carbon starvation are often seen to be associated with each other in the cascade of drought events leading to tree mortality (Adams et al., 2017).

Droughts, however, are very tougher to be defined, and thus a universally accepted drought definition considering both water-deficit intensity and duration is still lack (Buitink et al., 2021). Previous drought-related forest and agricultural studies, especially manipulation experiments, mainly focused on the effects of drought intensity (e.g., various levels of watering, such as very limited, limited, and optimum watering) on trees or plants (Allen et al., 2010; Yu et al., 2021), while less is known about the effects of drought duration on trees’ physiology, growth, and mortality. Actually, seasonal drought with longer duration occurs often in subtropical regions around the world. In terms of seasonal drought, drought duration is somewhat comparable to drought intensity. A long-lasting drought event may cause irreversible changes in plant physiology which are different from those found in relatively short-term severe droughts. For example, recurrent or short-term lasting drought events may allow trees to recover and even acclimate to water restriction (Gessler et al., 2020), and thus, permit them to survive in the long-term. However, longer term lasting drought events may strongly affect the recovery ability of trees. Currently, unexpected whole-season drought occurs more frequently in many regions around the world. For instance, since the beginning of the 21st century Europe already experienced severe drought summer of 2003, 2010, 2013, 2015, and 2018 (Hanel et al., 2018; Brunner et al., 2019). Therefore, mechanistic understanding of tree and forest responses to various drought duration is particularly critical for sustainably forest management under future climate change.

Theoretically, trees’ resilience and resistance to drought stress and the recovery and thus survival ability should be associated with the resource storage and availability. Non-structural carbohydrates (NSC = soluble sugars + starch) among other resources (e.g., nutrients, see Gessler et al., 2017) are known to contribute to tree resilience after stress (Li et al., 2002, 2008b). For many years, reserve storage was considered as a passive process resulting from an accumulation of resources when uptake and assimilation exceeded growth demand (Sala et al., 2012; Wiley and Helliker, 2012). An alternative hypothesis was proposed in which reserves storage would be rather an active process during the growing season and would act as a sink competing with other sinks (e.g., growth and reproduction) for available resources (Wiley and Helliker, 2012; Li et al., 2018a). Several studies investigated the NSC levels of trees during and at the end of the growing season following a growing season-long drought (Li et al., 2013; Schönbeck et al., 2018, 2020a,b). They found a drought-induced growth reduction but did not observe a drought-induced NSC decrease of trees, suggesting an active NSC storage under drought at the expense of growth (Wiley and Helliker, 2012; Li et al., 2018a). Recent evidence indicates that stress actively induces NSC transfer from aboveground tissues to roots stored (Kannenberg et al., 2018; Li et al., 2018a). In contrast, Li et al. (2018b) analyzed 27 case studies and found that drought decreased NSC concentration by 17.3% in roots, while it did not change NSC in aboveground tissues in the current season. To our knowledge, even less is known about the changes of NSC over-winter (post- vs. pre-winter) in trees previously stressed by drought.

Recently, it was proposed that nutrient addition (i.e., fertilization) will affect the fitness of trees under dry conditions, showing intensifying or mitigating effects on trees’ tolerance to drought (Kreuzwieser and Gessler, 2010; Gessler et al., 2017; Schönbeck et al., 2020b). Nitrogen (N) deficiency can increase the sensitivity of stomata to low leaf water potential (Radin and Ackerson, 1981; Ghashghaie and Saugier, 1989), which further increases the risk of drought-induced carbon starvation (McDowell, 2011). N itself is a main growth-limiting nutrient in temperate terrestrial ecosystems and is also a major component of Rubisco and other photosynthetic enzymes and structures which regulate the photosynthetic activity and thus carbon gain and the NSC level of trees in response to environmental factors, such as drought (Bond et al., 1999; Meng et al., 2016). Schönbeck et al. (2020a) found that negative effects of moderate drought intensity (but not of severe drought) could be compensated by increased nutrient availability in Scot pine saplings. In contrast, Jacobs et al. (2004) reported that fertilization with blended fertilizer impaired the root system development and drought avoidance ability of drought-stressed Douglas-fir seedlings. Similarly, Dziedek et al. (2016) found that nitrogen addition increased the drought sensitivity of saplings of several deciduous tree species (Dziedek et al., 2016). Despite these studies, there is a strong knowledge gap about drought and nutrient interaction and especially about whether and to what extent nutrient addition affects winter NSC consumption and thus growth recovery in the season following drought.

**Figure 1:**
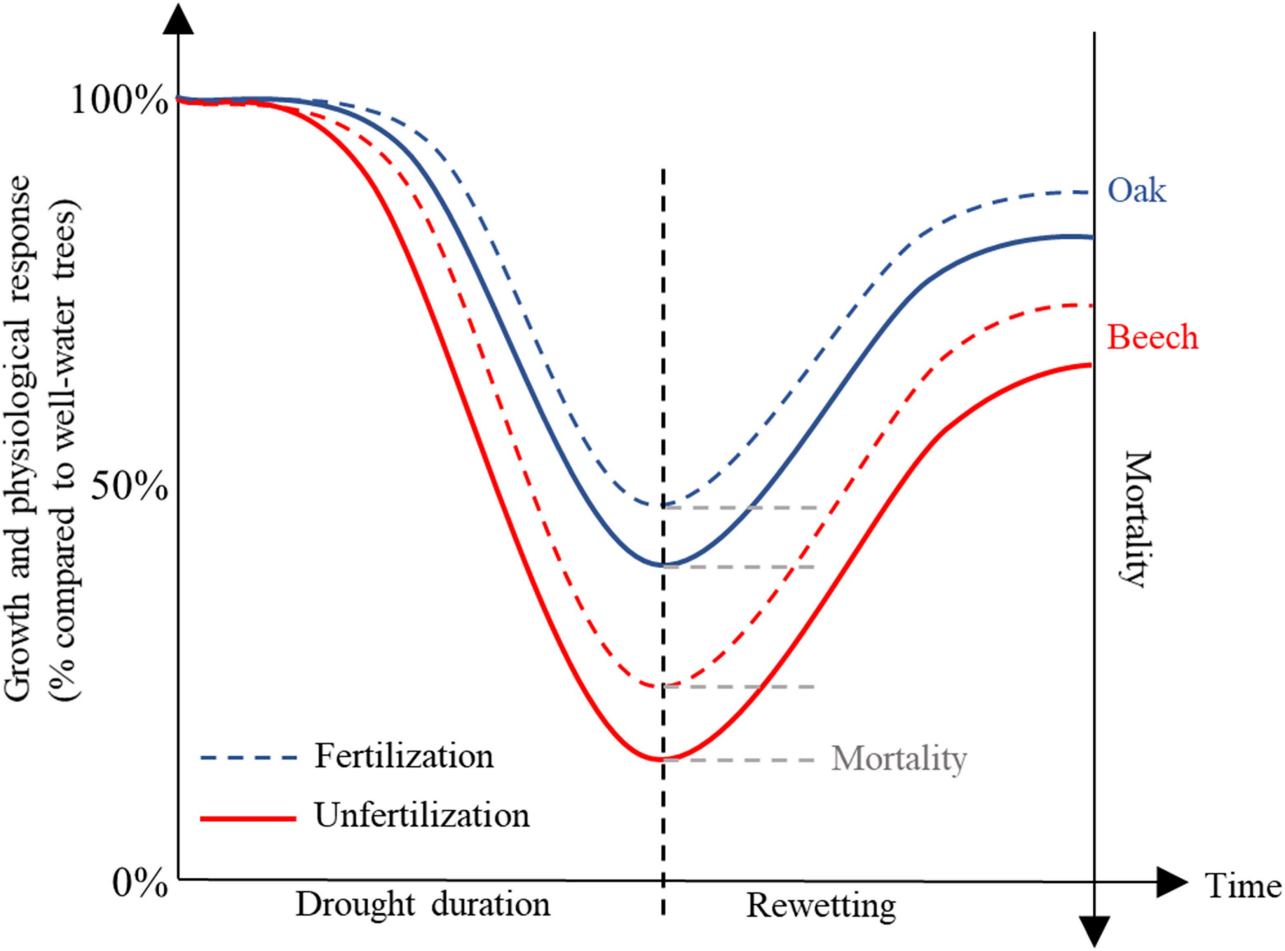
A conceptual model proposed to describe the physiological, growth, mortality, and recovery responses of *Quercus petraea* (oak) and *Fagus sylvatica* (beech) to increasing drought duration in combination with fertilization and after drought release.

The species, *Quercus petraea* (Matt.) Liebl. (*oak*) and *Fagus sylvatica* L. (beech), are two coexisting species in European forests. According to Ellenberg (2009), oak will become more competitive than beech at sites where as July temperatures increase to >18°C and precipitation decreases to <600 mm/year as a result of climate change. We proposed a conceptual model to describe the physiological, growth, survival, and recovery responses of these two species to increasing drought duration and after drought release (Figure 1), and therefore carried out a greenhouse experiment with saplings of these two species treated with various drought duration in combination with light fertilization (defined as <2 g N kg^−1^ dry soil), followed by rewetting (Figure 1). We measured leaf water potential, gas exchange rate, tissue NSC under various drought durations in combination with fertilization, and the recovery potential (incl. gas exchange, growth rate, and mortality) after rewetting, to test following hypotheses: (1) the availability of tissue NSC decreases with increasing drought duration due to decreased leaf water potential and photosynthesis; (2) this decreased pre-winter and post-winter (at the early beginning of next growing season) NSC availability leads to lower recovery ability of trees after rewetting; (3) fertilization mitigates the negative drought effects on trees as proposed by Gessler et al. (2017); and (4) beech is more sensitive than oak to the treatments as proposed by Ellenberg (2009).

## Materials and Methods

### Experimental Design and Treatments

The experiment was carried out in the greenhouse of the Swiss Federal Institute for Forest, Snow, and Landscape Research WSL (47°21′48″N, 8°27′23″E, 545 m a.s.l.), Birmensdorf, Switzerland. On February 27, 2018, 3-year-old sessile oak and European beech saplings (~30–40 cm in height) were planted into 10-liter plastic pots (26 cm in diameter). After transplanting into the pots, the plants were grown for 4 weeks under well-watered conditions (irrigation every 2–3 days) in the greenhouse to recover from the transplant shock. The cultivation soil consisted of semi-decomposed humus and commercial potting soil. The initial soil nitrogen and carbon contents were: 39 mg N kg^−1^ soil for NH_4_^+^-N, 573 mg N kg^−1^ soil for NO_3_^−^-N, 22.42% for soil C, and 0.82% for total soil N. The greenhouse temperature and humidity during the period of drought duration treatment are shown in Supplementary Figure A1.

A split-plot experimental design with three blocks was employed in this study. Each block was divided into two main plots, one of which was randomly assigned for oak (48 individuals) and the other one for beech (48 individuals; Supplementary Figure A2). Each plot was then divided into two sub-plots, one of which was randomly assigned for fertilization (24 individuals) and the other one for the non-fertilization treatment (ambient, 24 individuals; Supplementary Figure A2). Each sub-plot was further divided in to four sub-sub-plots (rows) randomly assigned for one of the four drought duration treatments (six individuals each). Therefore, a total of 288 plant individuals (144 individuals for each species) was included (Supplementary Figure A2).

Fertilization treatment may be ineffective when the application occurs during drought. Therefore, the fertilization treatment was conducted on 10 June 2018 (just prior to the drought duration treatment) when the soils were still moisture (Supplementary Figure A2). The fertilizer (Osmocote Exact 3-4M Standard, 7.0% NO_3_^−^-N, 9.0% NH_4_^+^-N, 9% P_2_O_5_, ICL, Suffolk, United Kingdom), equal to 1.68 g N kg^−1^ dry soil (0.945 g N kg^−1^ dry soil NH_4_^+^-N, 0.735 g N kg^−1^ dry soil NO_3_^−^-N) was added to each pot assigned for fertilization.

After the N-fertilization, plants were exposed to four drought duration treatments for 4 months in 2018 (Supplementary Figure A3). Plants in the well-watered treatment (D0) were watered thoroughly once a week. There were two moderate drought duration treatments, one of which was watered thoroughly biweekly (D1), and the other one was watered thoroughly once a month (D2; Supplementary Figure A3). The treatment of the longest drought duration in the present study was watered thoroughly once 2 months (D3; Supplementary Figure A3). We focused on the duration between two watering events and thus did not measure the soil water condition. After the harvest occurred on 1 October 2018, all remaining plants were treated (e.g., well-watering) in the same way until the end of the experiment (Supplementary Figure A3).

### Leaf Water Potential, Gas Exchange, Height Measurement, and Mortality Record

To detect the effects of drought duration, all measurements and sampling were always carried out directly before the next watering (Supplementary Figure A3).

Three plants from both nutrient and all drought treatments were randomly selected from each sub-sub-plot and three mature leaves from each selected plant were used for water potential and gas exchange measurements during the drought treatment in 2018. The midday leaf water potential (ψ_leaf_) was measured between 12:00 and 14:00 h on 5 August 2018 (Supplementary Figure A3), with a Scholander bomb (Model 600 pressure bomb; PMS Instrument Company, Albany, NY, United States). Net photosynthesis (A_leaf_) was measured on 6 August and 28 September 2018 (Supplementary Figure A3), and after rewetting in the following year on 24 June 2019, with a LiCor 6400 system (LI-COR, Lincoln, United States). A_leaf_ was measured at 400 μmol mol^−1^ CO_2_, 1,200 μmol m^2^ s^−1^ photosynthetically active radiation, *ca.* 65% relative humidity, and 25°C air temperature.

Plant height of each individual was measured on 30 May 2018 (initial height), on 1 October 2018 (end of the treatment), and on 8 October 2019 (after one recovery season; Supplementary Figure A3), and height increments were calculated.

### Harvest and Sampling

Destructive sampling (harvest) was conducted twice, one on 1 October 2018 as pre-winter samples, and the other one on 2 April 2019 as post-winter samples. One plant was randomly selected from each sub-sub-plot for each harvest time, and 48 individuals (24 individuals for each species; three for each nutrient and drought treatment) were harvested for each time (Supplementary Figure A2). The whole plant was harvested, the roots were carefully washed, and the plants were separated into leaves (Oct. 2018 only), shoots, and belowground tissue and separately sampled [leaves, shoots, mixed roots (i.e., fine and coarse roots)]. The samples were immediately put in an oven at 105°C for half an hour to stop the metabolic activity and then were dried at 65°C until stable weight. The dried samples were ground to fine powder, using a ball mill (MM 400; Retsch, Haan, Germany), for NSC analysis.

### Mortality Record

At the beginning of the experiment on 31 May 2018, there were six individuals for each treatment in each sub-sub-plot (Supplementary Figure A2). During the experiment period, one plant out of the six individuals in each sub-sub-plot was destructively harvested on each sampling date of 1 October 2018 and 2 April 2019 (Supplementary Figure A2). Thus, each species had, theoretically, four individuals (=6–2) left in each sub-sub-plot (Supplementary Figure A2), and each species had 12 individuals (4 × 3 blocks = 12) for each treatment across the three blocks available at the end of the recovery season 2019. However, in some sub-sub-plots all the four plants died by the end of 2019. Therefore, it was not possible to statistically analyze the treatment effects on the mortality. Instead, we recorded the total dead individuals (A) that did not sprout new leaves or shoots from any part of a plant during the recovery season across the three blocks and calculated the total mortality rate with (A/12) × 100%.

### Non-structural Carbohydrate Analysis

The NSC concentrations were measured according to the method by Wong (1990) modified by Hoch et al. (2002). NSC refers to the sum of mobile sugars (mainly glucose, fructose, and sucrose) and starch. First, 10–12 mg sample powder was boiled in 2 ml distilled water for half an hour. Then, 200 μl aliquot mixed with Invertase (Sigma-Aldrich, Buchs, Switzerland) were extracted to degrade sucrose to glucose and fructose. After centrifugation, glucose hexokinase and phosphogluconate isomerase (Sigma-Aldrich, Buchs, Switzerland) were added. The concentration of sugars was obtained as the total amount of glucose that was determined by 340 nm photometry (HR 7000, Hamilton, Rone, NE, United States) in a 96-well microplate photometer (Sigma-Aldrich, Buchs, Switzerland). 500 μl extract was taken from the sample aliquot and reacted with amyloglucosidase (Sigma-Aldrich, Buchs, Switzerland) for 15 h at 49°C, to break down starch to glucose, and to measure the total NSC concentration. The starch concentration was calculated as total NSC minus soluble sugars. NSC concentrations are expressed on a dry mass (d.m.) basis.

### Calculation and Statistical Analysis

The relative height increment rate (RHI) was calculated based on height measured on 30 May 2018 (A), on 10 October 2018 (B), and on 8 October 2019 (C), using [(B − A)/A] × 100% for the 2018 growth, and [(C − B)/B] × 100% for the 2019 recovery growth. The NSC concentration was calculated as the sum of the concentration of soluble sugars plus that of starch for each sample.

All data were checked for normality with the Kolmogorov–Smirnov test and for homogeneity of variance with Levene’s test. The effects of species, drought duration, N-fertilization, and their interactions on the parameters measured on each date were analyzed using linear mixed effect models, with block and main plot as random effects. Within each species, the effects of drought duration, N-fertilization, and their interactions on the parameters measured on each date were also analyzed using linear mixed effect models, with block and main plot as random effects, followed, if significant, by Tukey’s *post-hoc* test. Regression analysis was used to test the relationship between 2019 relative height increment rate and tissue NSC levels (both pre- and post-winter). All analyses are carried out by the package “LME” in R v.3.2.5 (R Core Team).

## Results

### Leaf Water Potential and Photosynthesis During Drought Duration Treatment

**Figure 2:**
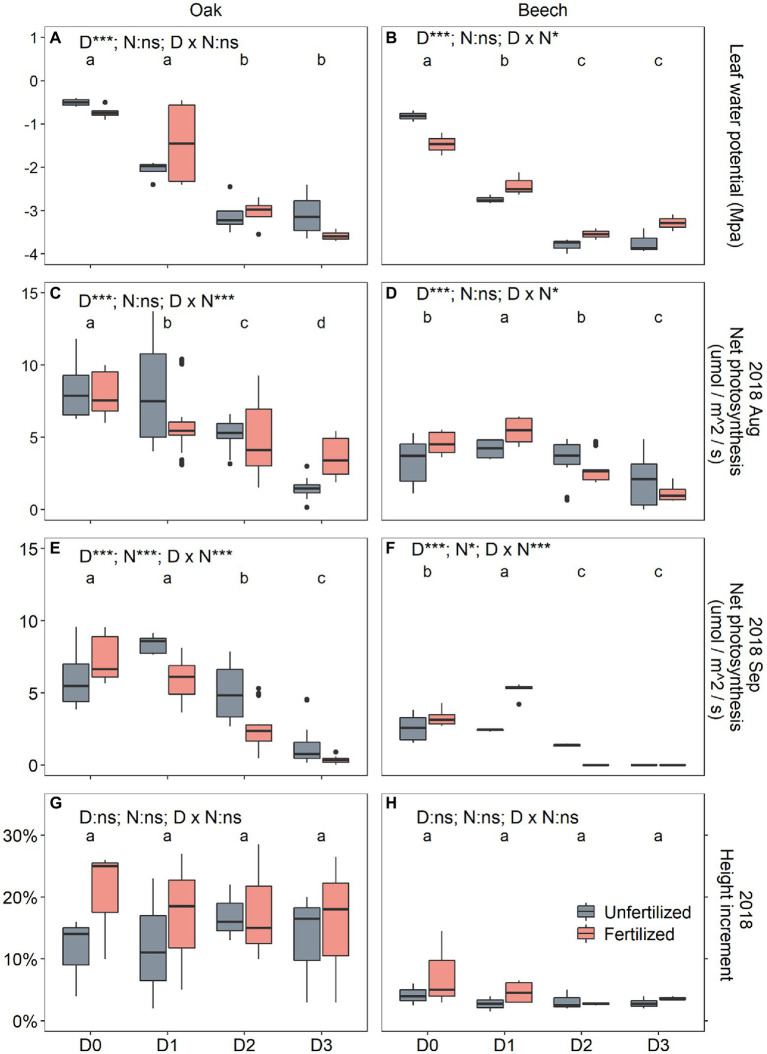
Midday water potential (ψ_leaf_) after 2 months of drought treatment on 5 August 2018 **(A,B)**, leaf net photosynthesis (*A*_leaf_) after two (6 August 2018; **C,D**) and four (28 September 2018; **E,F**) months of drought duration treatment in 2018, and the relative height increment (RHI; **G,H**) between 30 May (initial) and 1 October 2018 (after 4 months of drought treatment) for oak (*Quercus petraea*) and beech (*Fagus sylvatica*) saplings. D0 (watering weekly), D1 (watering biweekly), D2 (watering monthly), and D3 (watering bimonthly) represent the drought duration. Different letter indicates significant difference in parameters among D0, D1, D2, and D3.

Drought duration significantly decreased leaf water potential (Table 1; Figures 2A,B) in both species. A significant S × D interaction (*p* < 0.001; Table 1) indicated that the leaf water potential of the two species responded to drought duration significantly differently, showing a ψ_leaf_ order of D0 = D1 > D2 = D3 for oak (Figure 2A), and of D0 > D1 > D2 = D3 for beech (Figure 2B). Neither N-fertilization nor any interaction of N with other factors affected leaf water potential (Table 1). In beech, N-fertilization significantly decreased ψ_leaf_ in D0 plants (Figure 2B).

**Table 1:** Results of linear mixed models with species (S), drought duration (D), nitrogen fertilization (N) as factors, for water potential (ψ_leaf_), net photosynthetic rate during the drought duration treatment in 2018 and in the recovery growing season in 2019, relative height increment rate (RHI) in 2018 (drought) and 2019 (recovery).

	*df*	Leaf water potential		Photosynthesis (6 August 2018)		Photosynthesis (28 September 2018)		Photosynthesis (10 June 2019)		Relative height increment (2018)		Relative height increment (2019)	
		*F*	*p*	*F*	*p*	*F*	*p*	*F*	*p*	*F*	*p*	*F*	*p*
Species (S)	1	0.312	0.580	77.622	**<0.001**	132.315	**<0.001**	49.840	**<0.001**	17.219	<0.001	7.065	**<0.05**
D-duration (D)	3	13.826	**<0.001**	108.001	**<0.001**	233.175	**<0.001**	0.505	0.681	0.316	0.814	1.927	0.132
Nitrogen (N)	1	1.553	0.221	0.004	0.952	13.158	**<0.001**	0.310	0.580	2.332	0.138	0.381	0.539
S × D	3	6.982	**<0.001**	17.820	**<0.001**	9.902	**<0.001**	1.474	0.231	0.141	0.935	1.151	0.334
S × N	1	0.211	0.649	0.004	0.952	8.300	**<0.05**	0.972	0.328	0.569	0.457	0.112	0.739
D × N	3	0.781	0.513	9.317	**<0.001**	19.339	**<0.001**	0.381	0.767	0.561	0.645	1.759	0.162
S × D × N	3	0.047	0.986	11.456	**<0.001**	6.825	**<0.001**	1.006	0.397	0.290	0.833	0.720	0.543

Numbers of degrees of freedom (df), *F*- and *p*-values are given.

Bold text indicates a statistically significant difference with a *p*-value < 0.05.

Species, drought duration, N-fertilization, as well as their two-way and three-way interactions significantly affected the A_leaf_ of plants 2 months after the onset of the drought treatment on 6 August 2018 (except for non-effects of N and S × N interaction) and after 4 months on 28 September 2018 (Table 1). Oak’s A_leaf_ significantly decreased with drought duration for the two measurement dates (Figures 2C,E), while in beech A_leaf_ significantly increased from D0 to D1, and then decreased in D2 and D3 (Figures 2D,F) on both dates. N-fertilization seemed to decrease A_leaf_ of oak under D1 and D2 (Figures 2C,E), whereas it significantly increased A_leaf_ of beech under D1 (Figures 2D,F). Photosynthesis almost ceased in D3 plants after 4 months of treatment (Figures 2E,F), especially in beech (Figure 2F).

During the drought duration treatment, the relative height increment (RHI) differed significantly only between species (Table 1), showing higher RHI in oak than in beech (Figures 2G,H). Neither N and D nor their interactions affected RHI (Table 1; Figures 2G,H).

### Pre-winter NSC After Drought Duration Treatment

**Figure 3:**
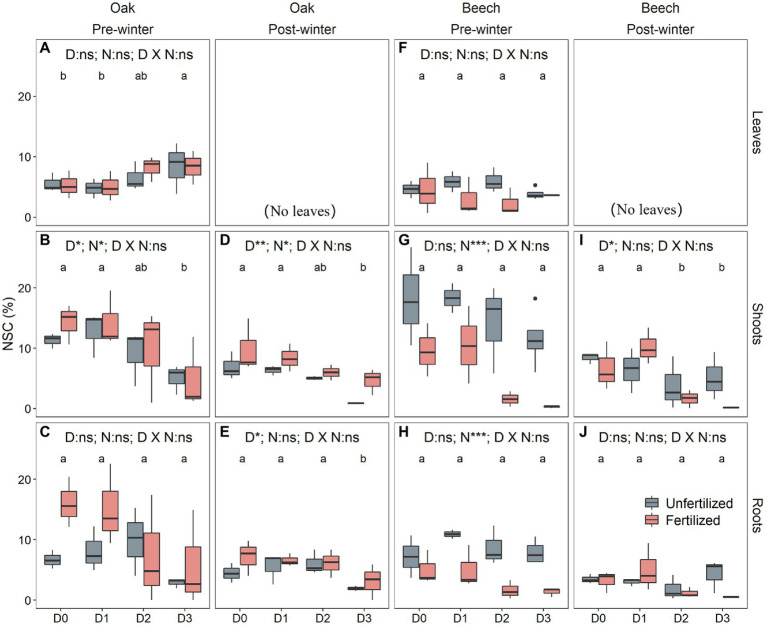
Non-structural carbohydrate (NSC) concentrations in leaves **(A,F)**, shoots **(B,D,G,I)**, and roots **(C,E,H,J)** of oak (*Quercus petraea*) **(A–E)** and beech (*Fagus sylvatica*) **(F–J)** saplings after 4 months of drought duration treatment in 2018 (pre-winter) **(A–E,F–H)** and before the growing season 2019 (post-winter) **(D–E,I–J)**. D0 (watering weekly), D1 (watering biweekly), D2 (watering monthly), and D3 (watering bimonthly) represent the drought duration. Different letters indicate significant differences in parameters among D0, D1, D2, and D3.

The pre-winter NSC levels in leaves differed significantly with species (Table 2). The shoot NSC levels, however, did not vary with species but were significantly affected by both drought treatment and N-fertilization (Table 2). The root NSC was significantly influenced by species and drought duration (Table 2). In addition, N-fertilization significantly interacted with species to affect the NSC levels in both shoots and roots (Table 2). Compared to the D0, D1, and D2 treatments, oaks in D3 had significantly higher leaf NSC levels (Figure 3A), mainly caused by higher sugar concentration (Supplementary Figure A4a), but significantly lower shoot NSC (Figure 3B) mainly resulting from significantly lower starch levels (Supplementary Figure A4e). The root NSC of oak tended to decrease with increasing drought duration (Figure 3C), due to both decreasing sugar and starch levels (Supplementary Figures A4c,f). For beech, both leaf and root NSC levels did not change with drought (Figures 3F,H) but shoot NSC decreased with increasing drought duration (Figure 3G) caused by both decreased sugar (Supplementary Figure A5b) and starch (Supplementary Figure A5l). N-fertilization significantly increased the shoot NSC in oak (Figure 3B), whereas it significantly decreased NSC levels in shoots (Figure 3G) and roots (Figure 3H) of beech.

**Table 2:** Results of linear mixed models with species (S), drought duration (D), nitrogen fertilization (N) as factors, for tissue NSC concentrations after the drought duration treatment in the growing season 2018 (pre-winter) and before the growing season 2019 (post-winter).

	*df*	Leaves		Shoots		Roots	
		*F*	*p*	*F*	*p*	*F*	*p*
*October 2018 (Pre-winter)*							
Species (S)	1	11.339	**<0.05**	0.003	0.958	6.207	**<0.05**
D-duration (D)	3	0.954	0.428	6.836	**<0.05**	2.768	**=0.05**
Nitrogen (N)	1	0.540	0.469	9.596	**<0.05**	0.276	0.603
S × D	3	1.864	0.158	0.228	0.876	1.100	0.365
S × N	1	1.932	0.175	16.091	**<0.001**	14.406	**<0.001**
D × N	3	0.156	0.925	0.303	0.823	2.002	0.136
S × D × N	3	0.869	0.468	0.076	0.973	0.495	0.689
*April 2019 (Post-winter)*							
Species (S)	1	(No leaves)		0.801	0.378	15.649	**<0.05**
D-duration (D)	3			12.511	**<0.001**	3.890	**<0.05**
Nitrogen (N)	1			0.627	0.435	0.227	0.637
S × D	3			1.122	0.356	2.451	0.083
S × N	1			5.977	**<0.05**	2.730	0.109
D × N	3			1.231	0.316	1.321	0.287
S × D × N	3			2.043	0.130	1.288	0.297

Numbers of degrees of freedom (df), *F*- and *p*-values are given.

Bold text indicates a statistically significant difference with a *p*-value < 0.05.

### Post-winter NSC After Drought Duration Treatment Last Growing Season

The post-winter NSC levels in both shoots and roots were significantly affected by drought duration treatment in the previous growing season (Table 2). The post-winter root NSC was species-dependent (*p* < 0.05; Table 2), and species interacted with N-fertilization to influence the post-winter shoot NSC (*p* < 0.05; Table 2). There was no direct effect of N on NSC for both species (Table 2). Both shoots and roots of D3 oak had significantly lower post-winter NSC levels compared to the other drought treatments (Figures 3D,E), mainly caused by both lower sugar and starch levels for shoots (Supplementary Figures A6a,c) and by lower sugar levels for roots (Supplementary Figure A6b). In beech only shoots of D2 and D3, due to both lower sugar and starch levels (Supplementary Figures A7a,c), showed significantly lower post-winter NSC levels compared to the other drought treatments (Figures 3I,J). N-fertilization significantly increased shoot NSC in oak (Figure 3D) but it had no effects on shoot (Figure 3I) and root NSC (Figure 3J) in beech.

### Changes in NSC Level Over-Winter

**Figure 4:**
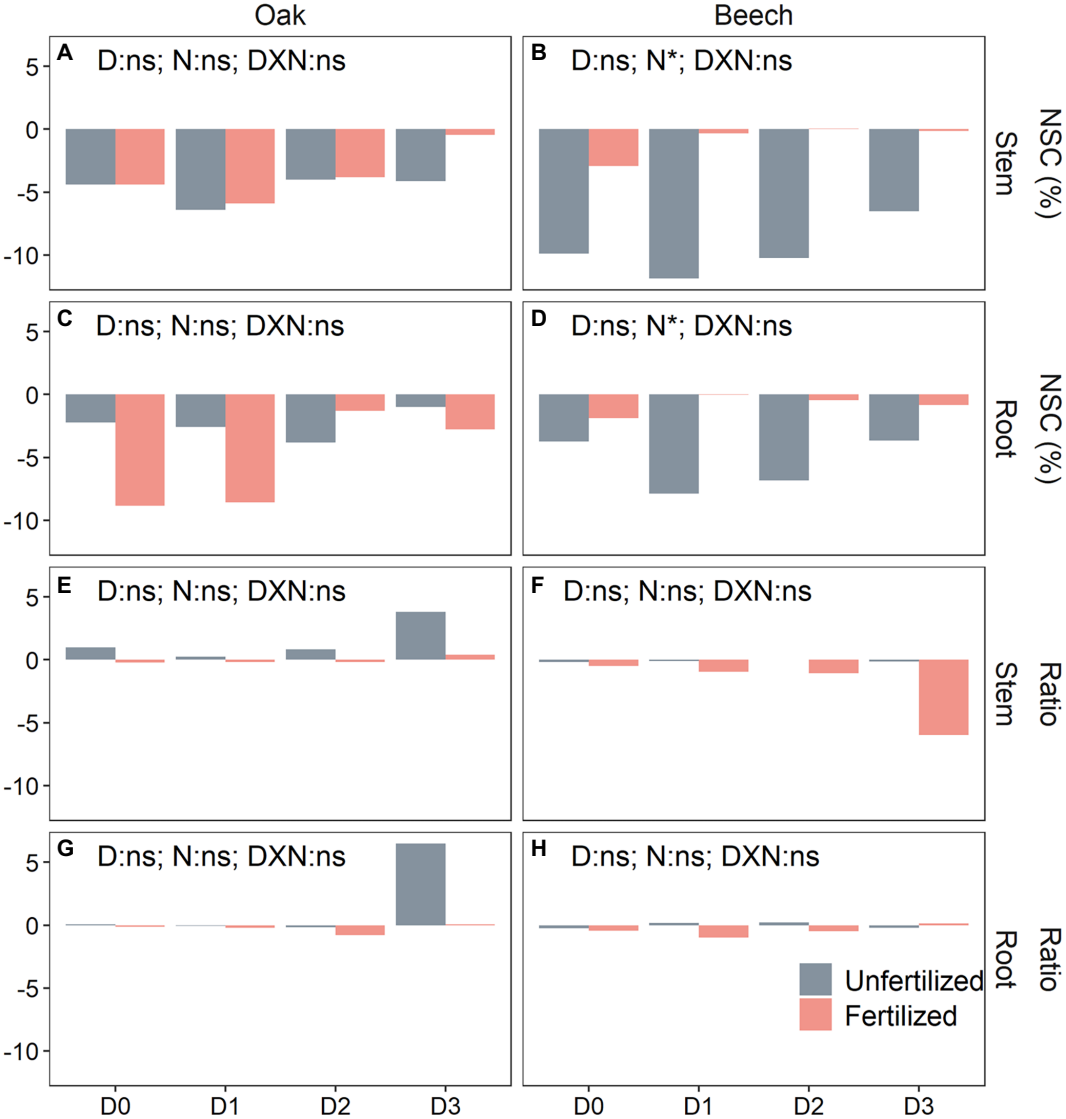
Over-winter changes in non-structural carbohydrate (NSC) concentrations **(A–D)** (post-winter values minus pre-winter values) and the sugar/starch ratio **(E–H)** in stem **(A,B,E,F)** and root **(C,D,G,H)** of oak (*Quercus petraea*) and beech (*Fagus sylvatica*) saplings treated with four drought durations during the previous growing season. D0 (watering weekly), D1 (watering biweekly), D2 (watering monthly), and D3 (watering bimonthly) represent the drought duration.

Only N-fertilization significantly affected the over-winter NSC consumption (post-winter level minus pre-winter level) in shoots but not in roots (Supplementary Table A1). Neither D nor N and their interactions changed the tissue NSC consumption in oak over-winter (Figures 4A,C), while N-fertilized beech significantly decreased the over-winter NSC consumption in both shoots and roots (Figures 4B,D). The changes in sugar/starch ratio were significantly influenced by both, species and N-fertilization (Supplementary Table A1). N-fertilization tended to decrease the sugar/starch ratio in beech, especially in shoots of D3 plants (Figures 4F,H), while the unfertilized oak saplings seemed to increase the tissue sugar/starch ratio over-winter, especially the D3 plants (Figures 4E,G).

### Recovery Responses to Past Drought and Rewetting

**Figure 5:**
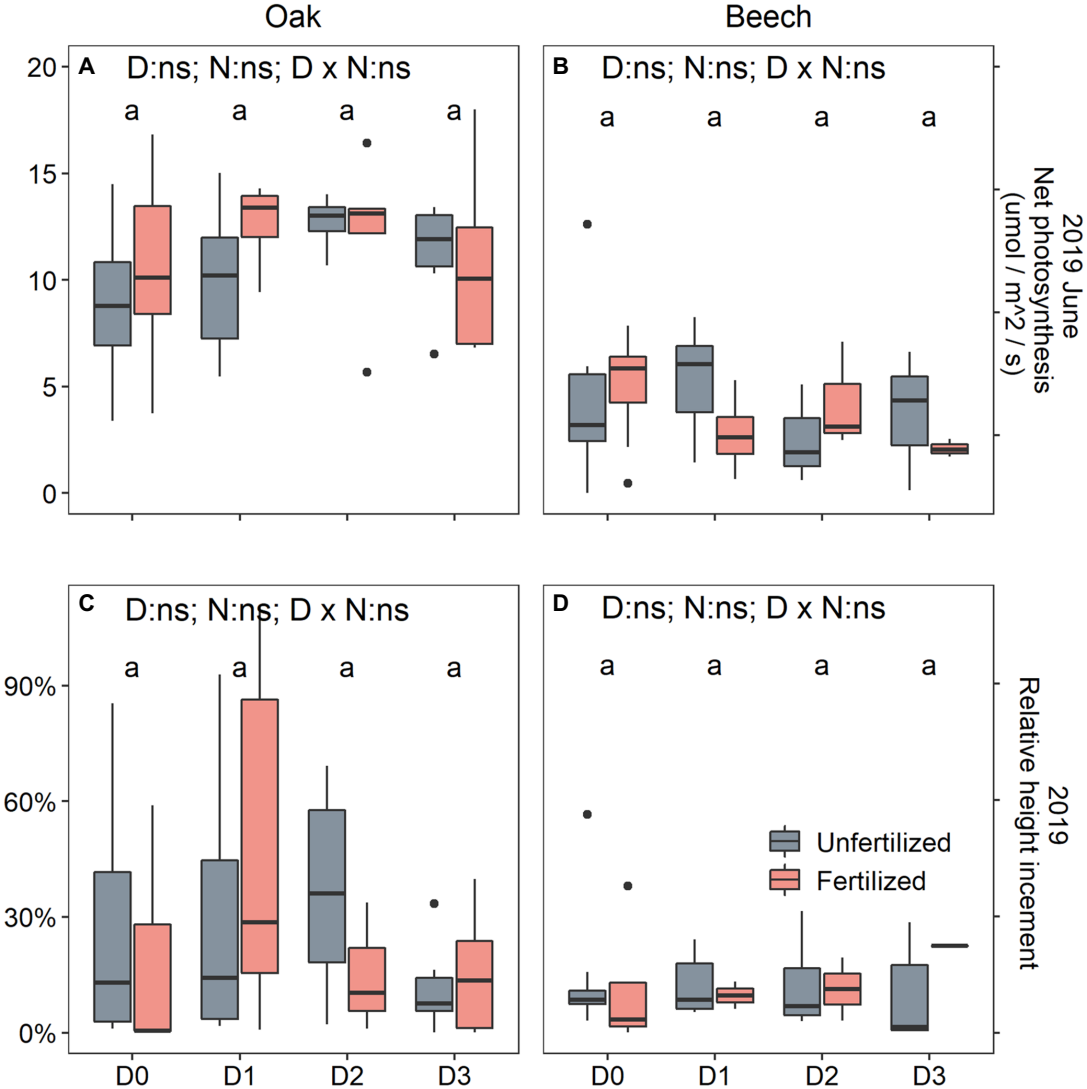
Leaf net photosynthesis (A_leaf_) **(A,B)** and relative height increment **(C,D)** of oak (*Quercus petraea*) **(A,C)** and beech (*Fagus sylvatica*) **(B,D)** saplings treated with four drought durations in 2018 and well-watered in 2019. D0 (watering weekly), D1 (watering biweekly), D2 (watering monthly), and D3 (watering bimonthly) represent the drought duration. Different letters indicate significant difference in parameters among D0, D1, D2, and D3.

After rewetting for one growing season, both the recovery photosynthesis measured on 10 June 2019 and recovery height growth measured on 8 October 2019 significantly responded to species only (Table 1), showing that both photosynthesis (Figures 5A vs. 4B) and height growth (Figures 5C vs. 4D) were greater in oak than in beech. Otherwise, previous season drought duration and N-fertilization treatment did not affect recovery responses of the two species (Figure 5).

**Figure 6:**
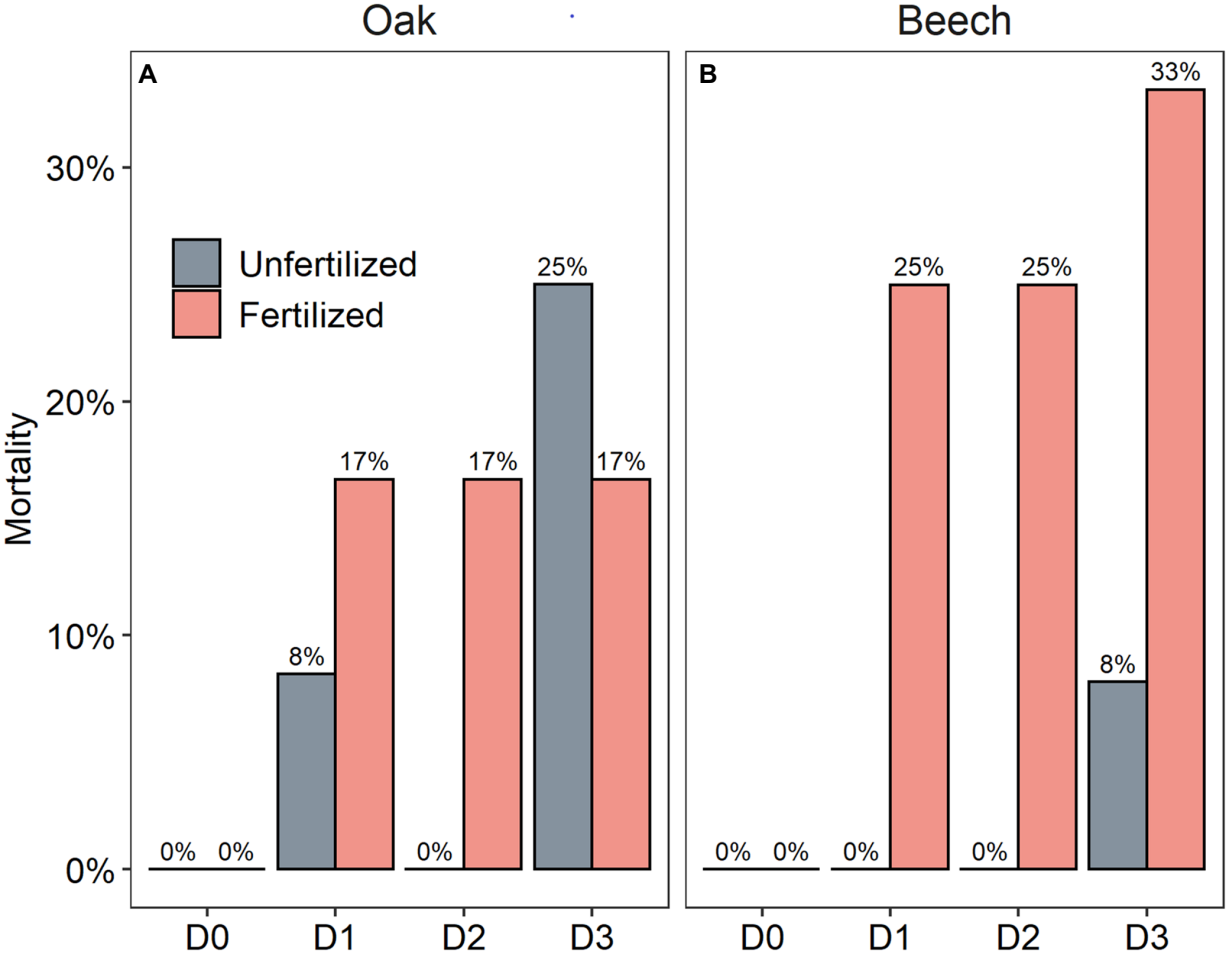
Mortality rate of oak (*Quercus petraea*) **(A)** and beech (*Fagus sylvatica*) **(B)** saplings at the end of a recovery growing season following a growing season with four drought duration treatment (D0 = watering weekly, D1 = watering biweekly, D2 = watering monthly, and D3 = watering bimonthly) combined with N-fertilization (1.68 g N kg^−1^ dry soil fertilized vs. unfertilized).

After a recovery growing season that followed the drought duration treatment in combination with N-fertilization, the mortality of beech was higher than oak, and the mortality rate seemed to show a tendency to increase with drought duration for the two species (Figure 6). Especially, the N-fertilized beech saplings had much higher mortality rate within each drought duration treatment category (Figure 6B).

## Discussion

With increasing drought duration, tree mortality increased (Figure 6), leaf water potential and photosynthesis decreased for the two species (Figure 2), which is similar to those results found in drought intensity experiments with trees (Schönbeck et al., 2018; Lauder et al., 2019; Archambeau et al., 2020; Schönbeck et al., 2020a; Ouyang et al., 2021). For instance, extreme drought was found to significantly decrease predawn water potential and net photosynthetic rates and to increase the mortality for both *Pinus sylvestris* (Schönbeck et al., 2020a) and *Quercus pubescens* saplings (Ouyang et al., 2021). Drought, both severe drought intensity and long drought duration, decreases soil water availability and plant leaf water potential, and thus results in stomatal closure to prevent transpiration exceeding root water uptake capacity, which caused declined photosynthesis and CO_2_ uptake (Li et al., 2020). Duan et al. (2019) found that severe drought intensity with short duration led to a stronger decrease in leaf water potential and photosynthesis of three tree species (*Syzygium rehderianum*, *Castanopsis chinensis*, and *Schima superba*) than moderate drought with longer duration. The water potential of *Robinia pseudoacacia* exhibited a linear decline with increasing drought duration, while *Quercus acutissima*’s water potential remained relatively stable during the first month of mild drought (Li et al., 2020), and thus, Li et al. (2020) concluded that the two tree species differ in their sensitivity to drought (Bhusal et al., 2021), which confirmed that *Quercus* species are anisohydric plants (Sade et al., 2012).

However, in spite of decreased photosynthesis (Figures 2C–F) and NSC levels (Figures 3B,C,G,H) with increasing drought duration, the relative tree height increment of the two species did not differ among the drought treatments in our study (Figures 2G,H). Li et al. (2013), Schönbeck et al. (2018, 2020a), and Ouyang et al. (2021) found that drought-stressed trees maintained relatively stable NSC levels at the expense of growth, implying an active process of NSC storage (Li et al., 2018a). For example, drought declined the growth but did not decrease tissue NSC level in *Quercus faginea* and *Pinus halepensis* (Sanz-Perez et al., 2009). The present study, however, seemed to support the view of Martinez-Vilalta et al. (2016) that NSC storage is mainly a passive process following the growth priority, because the growth did not vary with drought duration (Figures 2G,H) while the NSC levels in storage tissues, especially in shoots, decreased with increasing drought duration (Figures 3B–E,G). In this case, for example, the mortality (Figure 6B) of fertilized beech under D2 and D3 is thus mainly a result of carbon limitation that was confirmed by very low leaf photosynthetic rate (Figures 2D,F) and near-zero NSC level at the end season (Figures 3G,H). McDowell (2011) proposed that NSC concentrations can increase initially under drought due to the faster decline of growth than photosynthesis, but NSC concentrations may decline later on due to the prolonged suppression of photosynthesis and the utilization of stored C for meeting C demands especially under extreme drought.

Similar to results gained from most drought intensity experiments with trees (Li et al., 2013; Schönbeck et al., 2018, 2020a; Zhang et al., 2020; Ouyang et al., 2021), the present study found that the longer drought duration treatments (D2, D3) did not decrease the end-season leaf NSC (pre-winter) levels (Figures 3A,F), although the D2 and D3 treatment significantly decreased leaf photosynthesis of the two species (Figures 2C–F). This might be explained by the osmoregulation strategy of plants suffering from drought stress on the one hand (O’Brien et al., 2014; Dickman et al., 2015), and on the other hand, it may be a result of basipetal carbon translocation failure (Rowland et al., 2015), if the phloem function becomes impaired and carbon translocation gets limited or stopped by hydraulic failure caused by severe or long drought stress (Griffin-Nolan et al., 2021). In this case, lower NSC levels in the sink tissues of shoots, and especially roots, and thus carbon limitation may be expected. Recently, this expectation has been repeatedly confirmed in severe drought-stressed trees in controlled drought intensity experiment (e.g., Schönbeck et al., 2020a; Ouyang et al., 2021), and also in trees under longer drought duration (D2, D3) treatment in the present study (Figures 3B,C,G,H). Therefore, it may be speculated that a hydraulic failure induced carbon limitation seems to be the physiological mechanism underlying the high mortality of beech saplings, particularly the N-fertilized D2 and D3 beech (Figure 6B) which had very low end-season shoot and root NSC levels close to zero (Figures 3G,H). These results also seem to exclude that limited sink activity, for example, in root tissues as a result of drought is responsible for reduced sugar transport from the leaves to the sink tissues as in that case increased NSC concentrations are to be expected in both, roots and shoots (Hagedorn et al., 2016; Gessler and Grossiord, 2019).

We found that the responses of end-season NSC level to drought duration seemed to be both species- and tissue type-dependent (Figures 3A,B,F–H). For instance, leaf NSC increased (Figure 3A) but shoot NSC decreased (Figure 3B) in oak with increased drought duration, while they did not change in beech (Figures 3F,G). Similar to beech, root NSC reserve of aspen (*Populus tremuloides*) seedlings did not change over a 3-month period of severe drought (Galvez et al., 2011). However, moderate drought was found to increase NSC in stems and roots of *Q. pubescens* saplings (Ouyang et al., 2021). Experiments with more vs. less precipitation found that extreme drought (no irrigation for two consecutive years) reduced shoot and root NSC, whereas intermediate drought levels did not affect shoot and root NSC for *P. sylvestris* saplings (Schönbeck et al., 2020a).

Less is known about winter NSC consumption of trees previously exposed to drought of various intensities or duration. Trees, as exemplified by the deciduous species in the present study, consume NSC storage for maintenance respiration over-winter (Sperling et al., 2015). Therefore, we found that the post-winter NSC levels were lower than the pre-winter level in each tissue for both species (Figures 3B vs. 3D; Figures 3C vs. 3E; Figures 3G vs. 3I; Figures 3H vs. 3J). The winter temperature was beyond 5°C in the greenhouse of the present study (Supplementary Figure A1), but even near-freezing winter temperatures were found to significantly increase stem respiration by 10–170% in 13 out of 15 species studied in the western US, according to Sperling et al. (2015). Sperling et al. (2015) further calculated that “frost-induced respiration accelerated stem NSC consumption by 8.4 mg (glucose eq.) cm^−3^ year^−1^ on average (cm^−3^ stem wood basis) in the western US, a level of depletion that may continue to significantly affect spring NSC availability.” This is agreement with findings that in temperate deciduous trees, tissue NSC concentrations decline during winter dormancy. This decrease is more pronounced in stem than in roots as observed for aspen (*Populus grandidentata*) and oak (*Quercus rubra*; Gough et al., 2010). The present study, for the first time, indicated that the over-winter NSC consumption was not affected by drought duration for the two species but it was significantly decreased by N-fertilization for beech across the four drought treatments (Supplementary Table A1; Figures 4A–D). This result may indicate on the one hand a common response of winter NSC consumption of tree species that is independent on the previously imposed drought duration. On the other hand, our results suggest a species-specific sensitivity of winter NSC consumption to other environmental change, such as nutrient availability. We can only speculate why the NSC consumption was lower in fertilized beech but it is known that free amino acids and soluble proteins can increase stress resistance of beech (Stajner et al., 2013). Thus, an increased N availability might reduce stress-induced respiration in this species under winter temperature conditions (Supplementary Figure A1).

The over-winter changes (post-winter vs. pre-winter) in the sugar/starch ratio (Figures 4E–H) indicated that starch to sugar conversion occurred in oak saplings (Figures 4E,G; Supplementary Figures A4, A6), whereas a strong sugar consumption and depletion were the main reasons for decreased tissue sugar/starch ratio in beech saplings (Figures 4F–H; Supplementary Figures A5, A7). Similarly, starch concentrations were reduced and soluble sugars increased in *Prunus dulcis* during winter, and the NSC concentration were only slight reduced (Sperling et al., 2019). In winter, increased sugar concentrations in the xylem are important to avoid or reduce the number of freeze–thaw embolization cycles, because sugars increase the osmotic potential of xylem and thus lowering its freezing point (Sauter et al., 1973; Thierry et al., 2004; Li et al., 2018b).

Previous season drought duration treatment did not affect photosynthesis of the two species after rewetting in the next year (Table 1). The decreased photosynthesis determined in the longer duration drought treatments in 2018 (Figures 2C–F) recovered and all treatments showed the same level of photosynthesis in June 2019 (Figures 5A,B). This recovery indicates that there is no legacy of previous drought duration on photosynthetic carbon assimilation. Previous studies found that drought stress can result in incomplete and lagged growth recovery (Anderegg et al., 2013; Pederson et al., 2014; Huang et al., 2018). Extreme drought caused drought legacy response with reduced growth for deep−rooted forests for up to 4 years (Wu et al., 2018), and negative drought legacy was found to last about 1 year for different plant functional types in Tibetan Plateau (Li et al., 2020). However, fast recovery of carbon acquisition and allocation to different plant organs after drought release was also observed in different tree species (Hagedorn et al., 2016; Joseph et al., 2020). In the present study, small saplings with large plasticity may be one reason for the quick recovery after rewetting, leading to a lack of legacy of past drought. Similar results have been observed in poplar (*Populus tremula*) saplings under water-deficit conditions (Lemaire et al., 2021). In addition, the longest drought duration (2 months) applied here may be still not long (or severe) enough to impair the physiological processes in the longer term. For example, an open top chamber experiment with 40 cm soil depth but without any watering for 2 years resulted only in a mortality rate of 60% for *P. sylvestris* (Schönbeck et al., 2020a) and 50% for *Q. pubescens* saplings (Ouyang et al., 2021). In line with the findings of Schönbeck et al. (2020a) for pine, we found a fertilization-induced higher mortality rate for beech but not for oak saplings (Figure 6). Ouyang et al. (2021) showed a lower mortality rate (32%) in fertilized compared to non-fertilized (50%) *Q. pubescens* saplings under extreme drought. In oak species, fertilization might thus not enhance drought effects but genus and species-specific mechanism still need to be elucidated.

The height growth of the two species was not correlated with shoot NSC storage but significantly positively correlated with both pre-winter (*p* = 0.07) and post-winter (*p* = 0.01) root storage (Supplementary Figure A8). The small values of *R*^2^ (Supplementary Figure A8) suggest that root NSC is not the only or the most important factor determining the recovery growth. However, this result (Supplementary Figure A8) in conjunction with low photosynthetic rate (Figures 2C–F), low pre- and post-winter NSC levels in both shoots and roots of D2 and D3 saplings suggests a sink carbon limitation that determines the high mortality rate of D2 and D3 saplings for the two species, particularly for beech (Figure 6). This result supports a recent hypothesis that the climatic alpine treeline is determined by a winter root carbon limitation proposed by Li et al. (2018a). Our result is also supported by data for *P. sylvestris* (Schönbeck et al., 2020a) and *Q. pubescens* saplings (Ouyang et al., 2021) under extreme drought that also showed low root NSC. Indeed, root carbon shortage has been widely found in various tree and shrub species in stressed conditions recently (Shi et al., 2006; Li et al., 2008a,b; Genet et al., 2011; Zhu et al., 2012a,b; Ouyang et al., 2021; Wang et al., 2021).

Interactions between drought duration and N-fertilization were found only for gas exchange rate during the treatment period (Table 1), indicating that the effects of N-fertilization vary in direction (or magnitude) with drought duration only for photosynthesis but not on other parameters studied (Table 2 and Supplementary Table A1). Schönbeck et al. (2020a) found that a mitigating effect of N-fertilization on the negative drought effects on *P. sylvestris* saplings occurred only when the drought stress was relatively mild. On the other hand, the effects of fertilization on drought may also be dependent upon the initial soil fertility, and a mitigating effect of fertilization on drought may not be expected in nutrient-rich soils (Kleczewski et al., 2010). For example, the soil used in the present study had a total soil N content of 0.82% with 39 mg N kg^−1^ soil for NH_4_^+^-N and 573 mg N kg^−1^soil for NO_3_^−^-N, which, probably, led to a non-significant effect of fertilization. In a summer drought experiment (no rainfall for two summer months during two consecutive years) it was found that the negative effects of drought on beech growth were amplified by N-fertilization (Dziedek et al., 2016), which is similar to our results that drought-induced mortality of beech was amplified by N-fertilization (Figure 6B). Theoretically, increases in N availability may promote the formation of xylem structures that transport water more efficiently in humid conditions (Borghetti et al., 2017) but may also easily lead to xylem embolism—due to larger cross section and bigger tracheids or vessels—in dry conditions, and therefore, further studies are needed to clarify the N-fertilization effects (e.g., addition rate and amount and frequency) in relation to drought intensity or duration not only on seedlings and saplings but also adult trees.

## Conclusion

In line with our hypothesis 1, we found that longer drought duration decreased the physiological performance (e.g., leaf water potential, photosynthetic capacity, and NSC levels) but not the growth rate. This result suggest that growth is a higher priority than resource storage for the saplings of the two species stressed by long-lasting drought below a certain threshold, as recently proposed by Song et al. (2022). Previous growing season drought seems to not affect the tissue NSC consumption over-winter, and the post-winter root NSC level plays a more important role in determining the growth and survival for both species (see our hypothesis 2 in introduction), suggesting a root carbon limitation in severe drought-stressed saplings, particularly for beech. In line with recent findings (Schönbeck et al., 2018, 2020a; Ouyang et al., 2021), N-fertilization did not play a role to mitigating the negative drought effects on saplings of the two species (hypothesis 3). Compared to oak, beech had lower levels of physiological parameters and growth but showed higher winter NSC consumption and especially higher mortality rate with increasing drought duration in combination with fertilization, indicating that beech is more sensitive to drought and N deposition (hypothesis 4). The present study, in a physiological perspective, experimentally confirmed the view of Ellenberg (2009) that the European beech, compared to oak, may be more strongly affected by future environmental changes.

## Data Availability Statement

The raw data supporting the conclusions of this article will be made available by the authors, without undue reservation.

## Author Contributions

M-HL designed the experiment. SO conducted the experiment and collected the samples. RN and XW conducted the data analysis. YY wrote the first draft of the manuscript. AG, HH, ZW, and M-HL put forward some constructive suggestions for the manuscript. All authors contributed to the article and approved the submitted version.

## Funding

This study was supported by the Swiss National Fund (31003A_157126/1) and China Scholarship Council (CSC) (201906620095). Open access funding provided by WSL - Swiss Federal Institute for Forest, Snow and Landscape Research.

## Conflict of Interest

The authors declare that the research was conducted in the absence of any commercial or financial relationships that could be construed as a potential conflict of interest.

## Publisher’s Note

All claims expressed in this article are solely those of the authors and do not necessarily represent those of their affiliated organizations, or those of the publisher, the editors and the reviewers. Any product that may be evaluated in this article, or claim that may be made by its manufacturer, is not guaranteed or endorsed by the publisher.
