# Supplementary material for: Root Carbon Resources Determine Survival and Growth of Young Trees Under Long Drought in Combination With Fertilization
Source: Front Plant Sci. 2022 Jun 3;13:929855. doi: 10.3389/fpls.2022.929855 (PMC9204053; doi:10.3389/fpls.2022.929855)
Supplement: Supplementary file 1 [file Data_Sheet_1.docx]

**Table A. 1** Results of linear mixed models for the changes of NSC and ratio of sugar and starch changes overwintering under different treatments in shoots and roots tissues. No. of degree freedom, F and P-values are given for different factors as species (S), drought treatments (D), nitrogen treatments (N).

|  | *df* | Changes in NSC | | | | | | Changes in sugar/starch ratio | | | | |
| --- | --- | --- | --- | --- | --- | --- | --- | --- | --- | --- | --- | --- |
|  |  | Shoots | |  | Roots | |  | Shoots | |  | Roots | |
|  |  | *F* | *P* |  | *F* | *P* |  | *F* | *P* |  | *F* | *P* |
| Species (S) | 1 | 0.581 | 0.463 |  | 0.197 | 0.666 |  | 4.872 | **=0.05** |  | 1.271 | 0.286 |
| D-duration (D) | 3 | 1.088 | 0.398 |  | 0.529 | 0.673 |  | 0.070 | 0.975 |  | 1.422 | 0.294 |
| Nitrogen (N) | 1 | 13.058 | **<0.05** |  | 0.284 | 0.606 |  | 4.505 | **=0.05** |  | 2.069 | 0.181 |


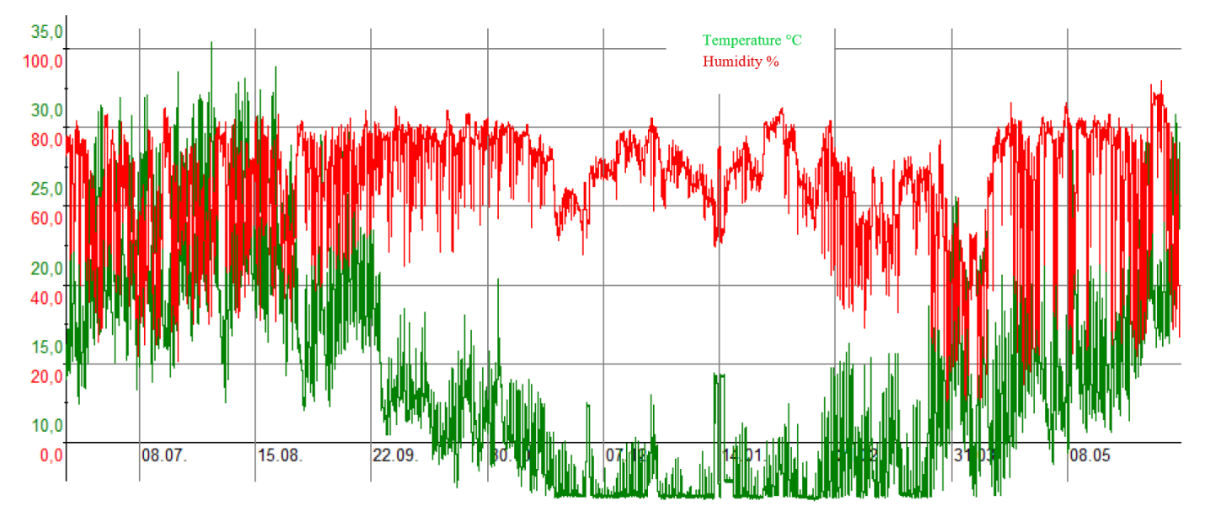


Fig. A. 1 Daily temperature and humidity during the period of drought duration treatment in the greenhouse.


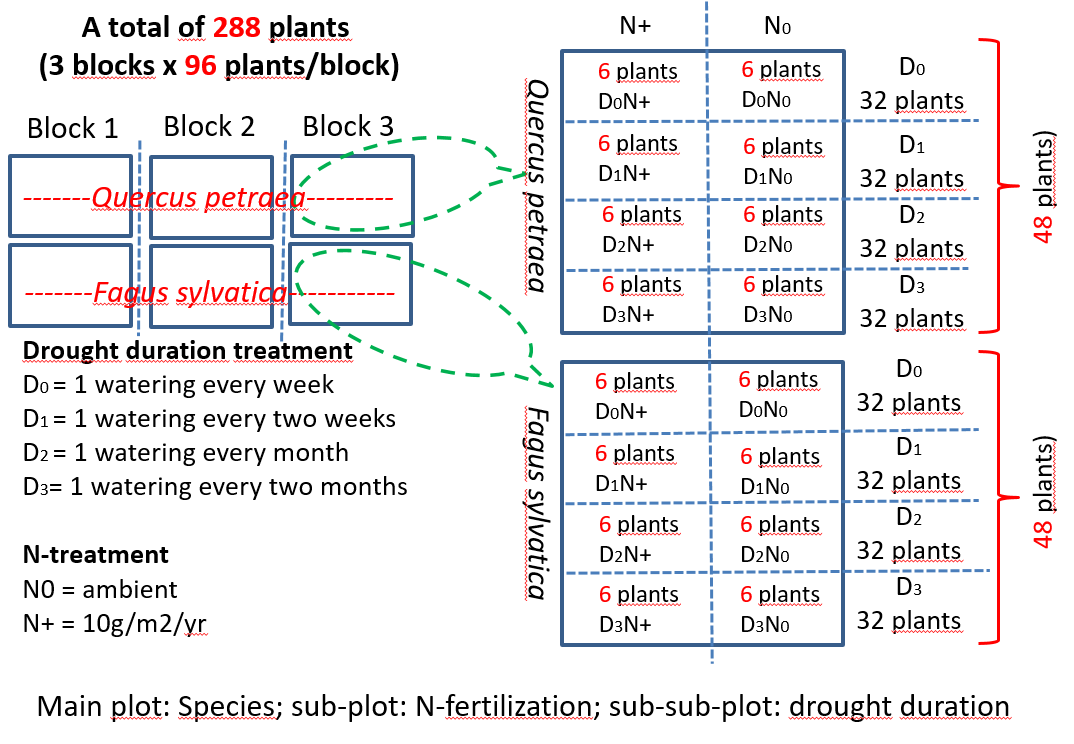


Fig. A. 2 Schematic plan of the experimental design.


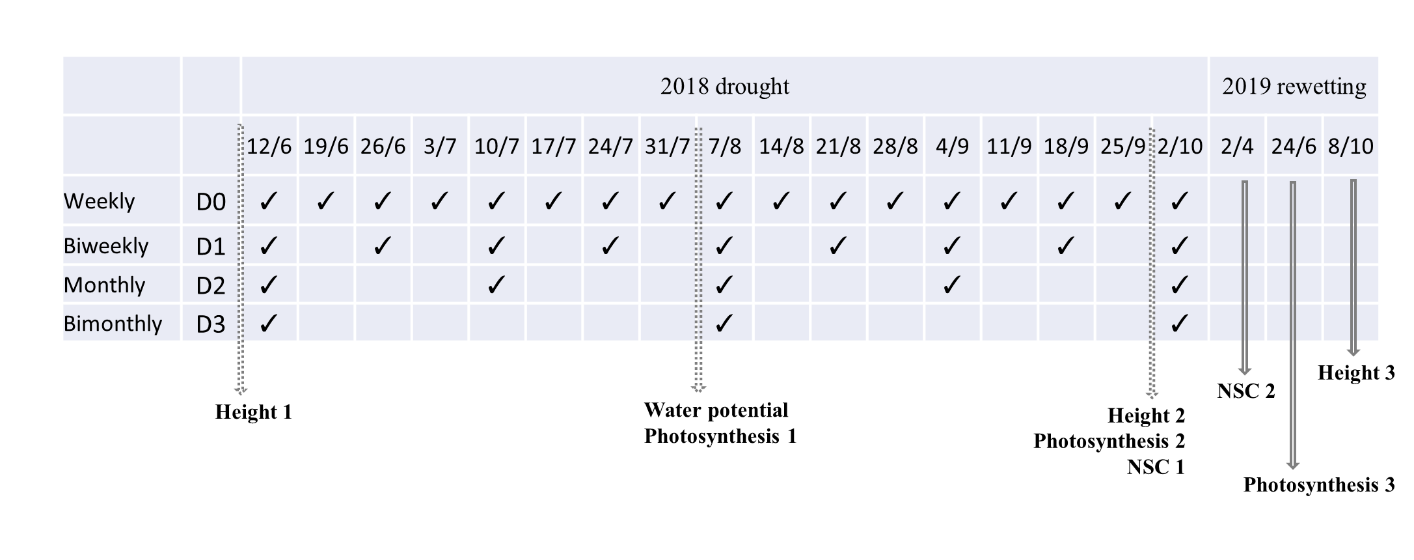


Fig. A. 3 Schematic representation of the experiment indicating the watering date and major measurements times in the drought year (2018) and rewetting year (2019).


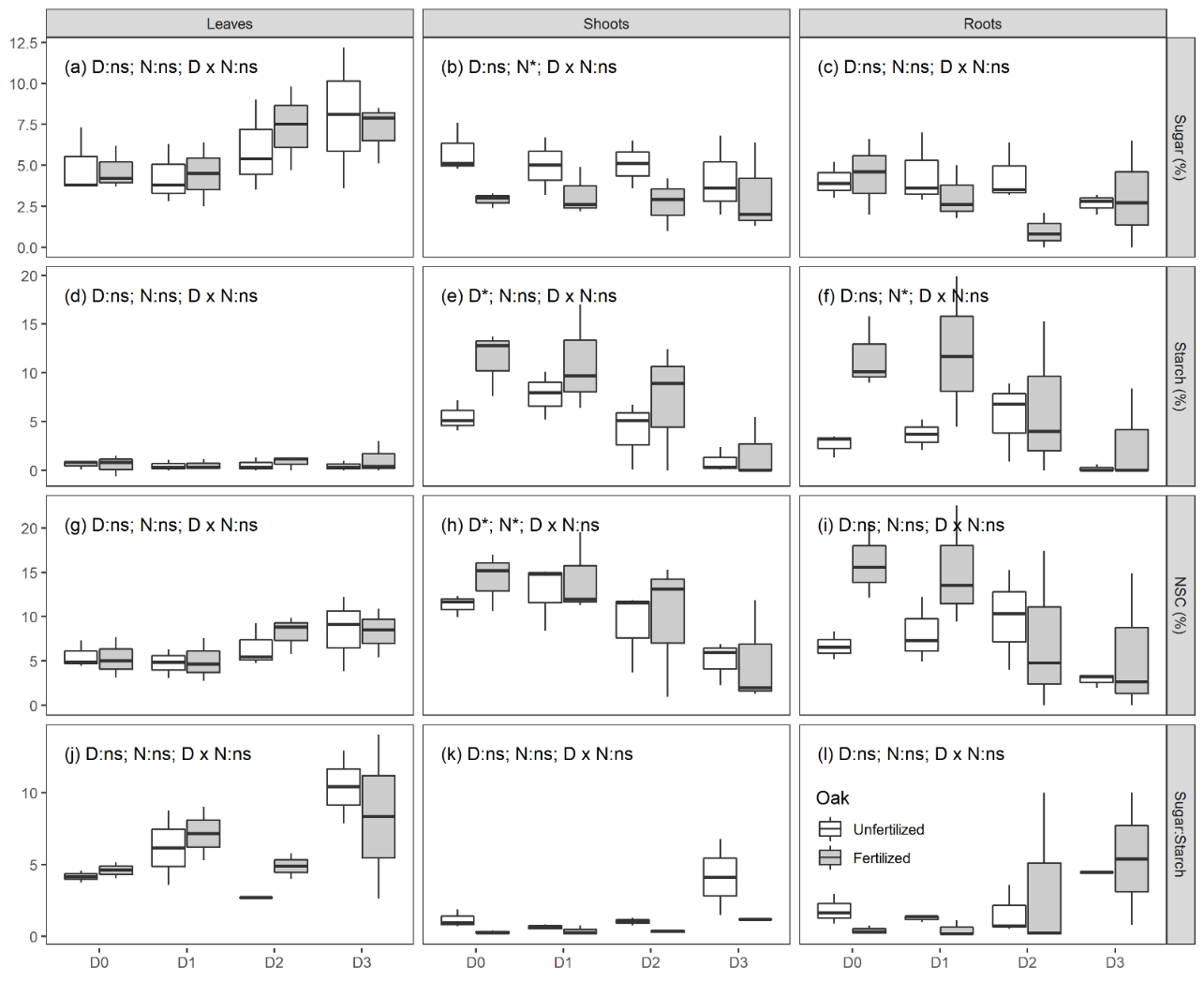


Fig. A. 4 Pre-winter levels of soluble sugars, starch, and non-structural carbohydrate (NSC), as well as the ratio of sugars to starch in leaves, stems and roots of oak (*Quercus petraea*) after the 2018 growing season treatment with four drought durations in combination with N-fertilization.


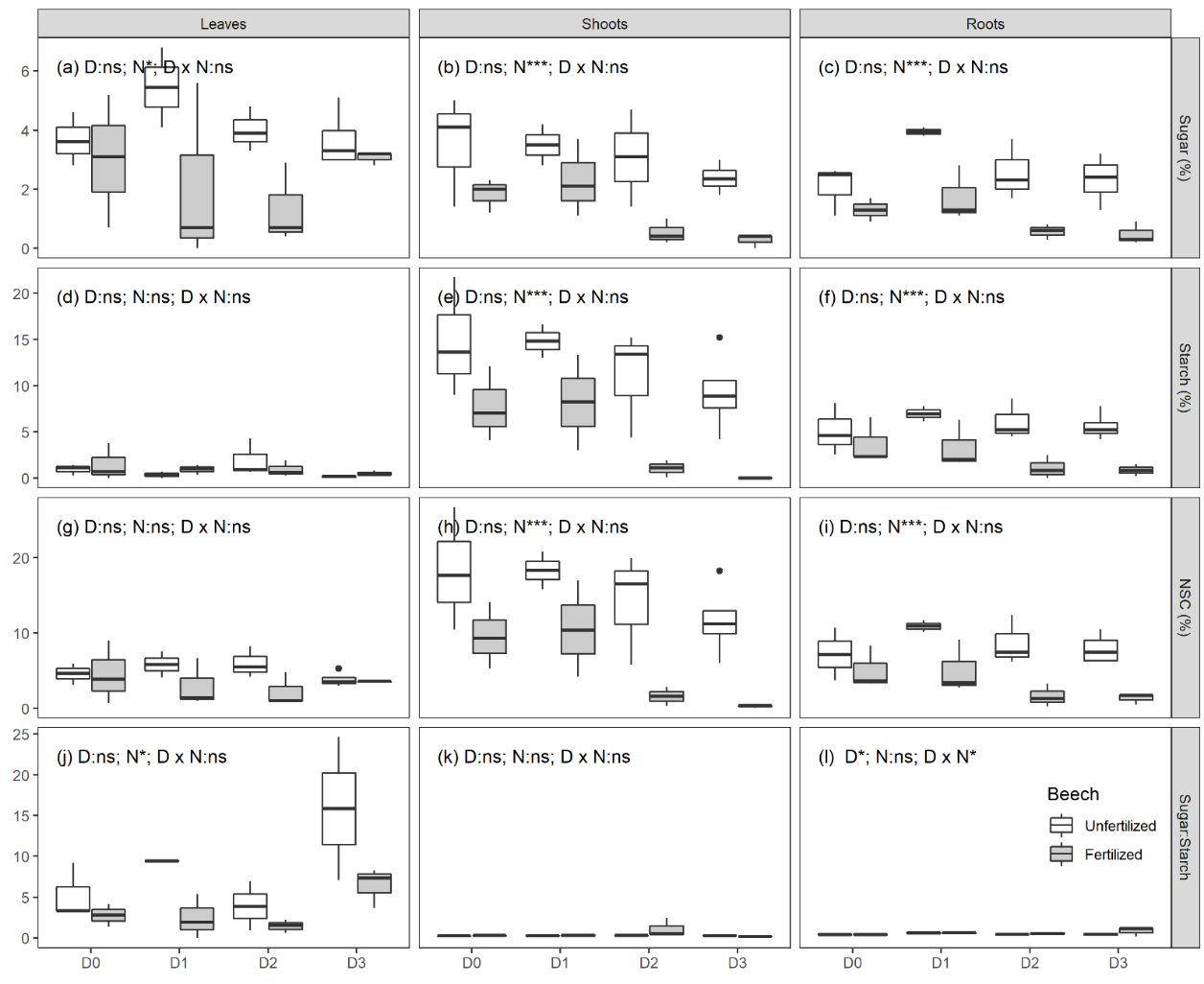


Fig. A. 5 Pre-winter levels of soluble sugars, starch, and non-structural carbohydrate (NSC), as well as the ratio of sugars to starch in leaves, stems and roots of beech (*Fagus sylvatica*) after the 2018 growing season treatment with four drought durations in combination with N-fertilization.


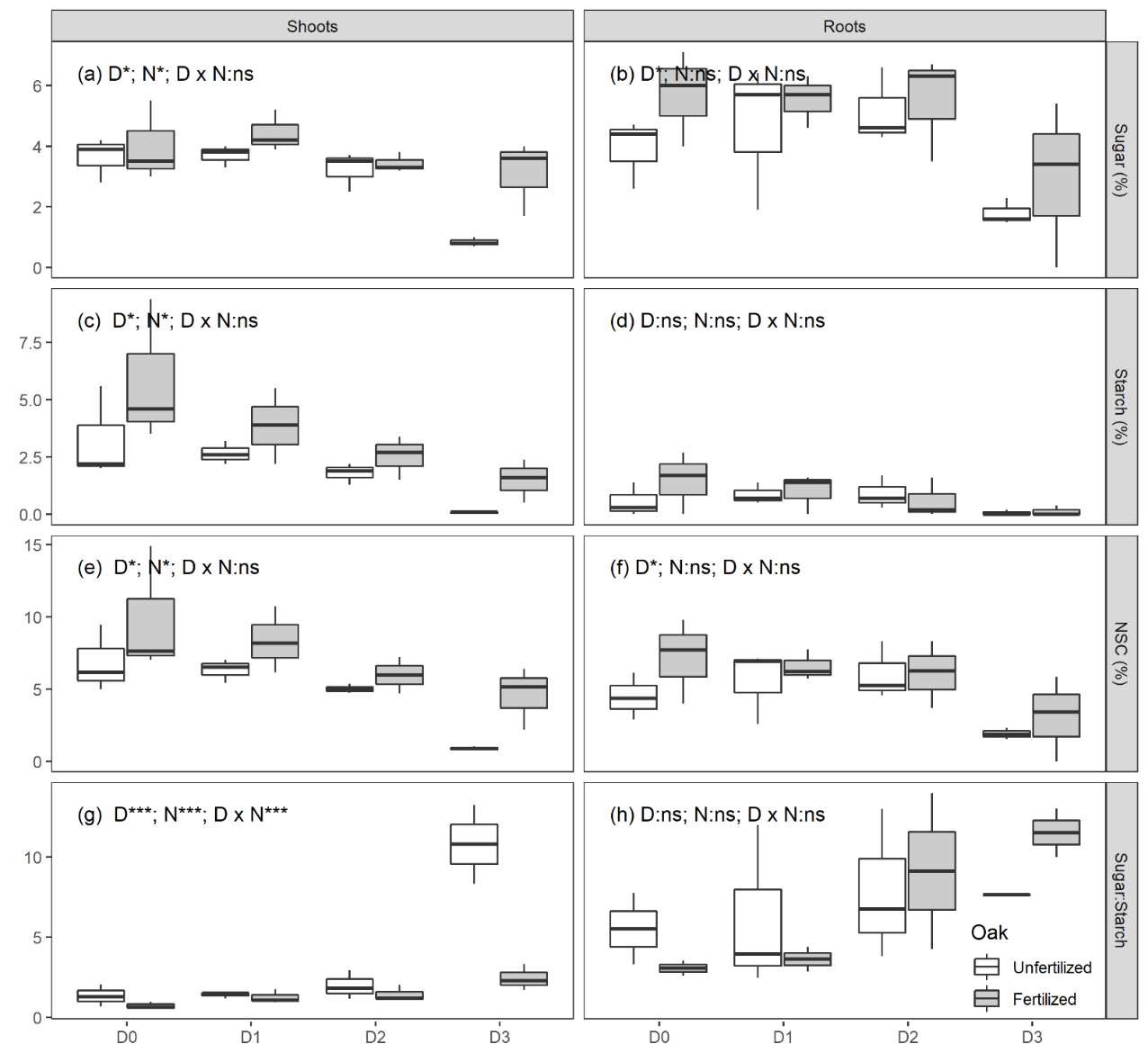


Fig. A. 6 Post-winter levels of soluble sugars, starch, and non-structural carbohydrate (NSC), as well as the ratio of sugars to starch in stems and roots of oak (*Quercus petraea*) after the 2018 growing season treatment with four drought durations in combination with N-fertilization.


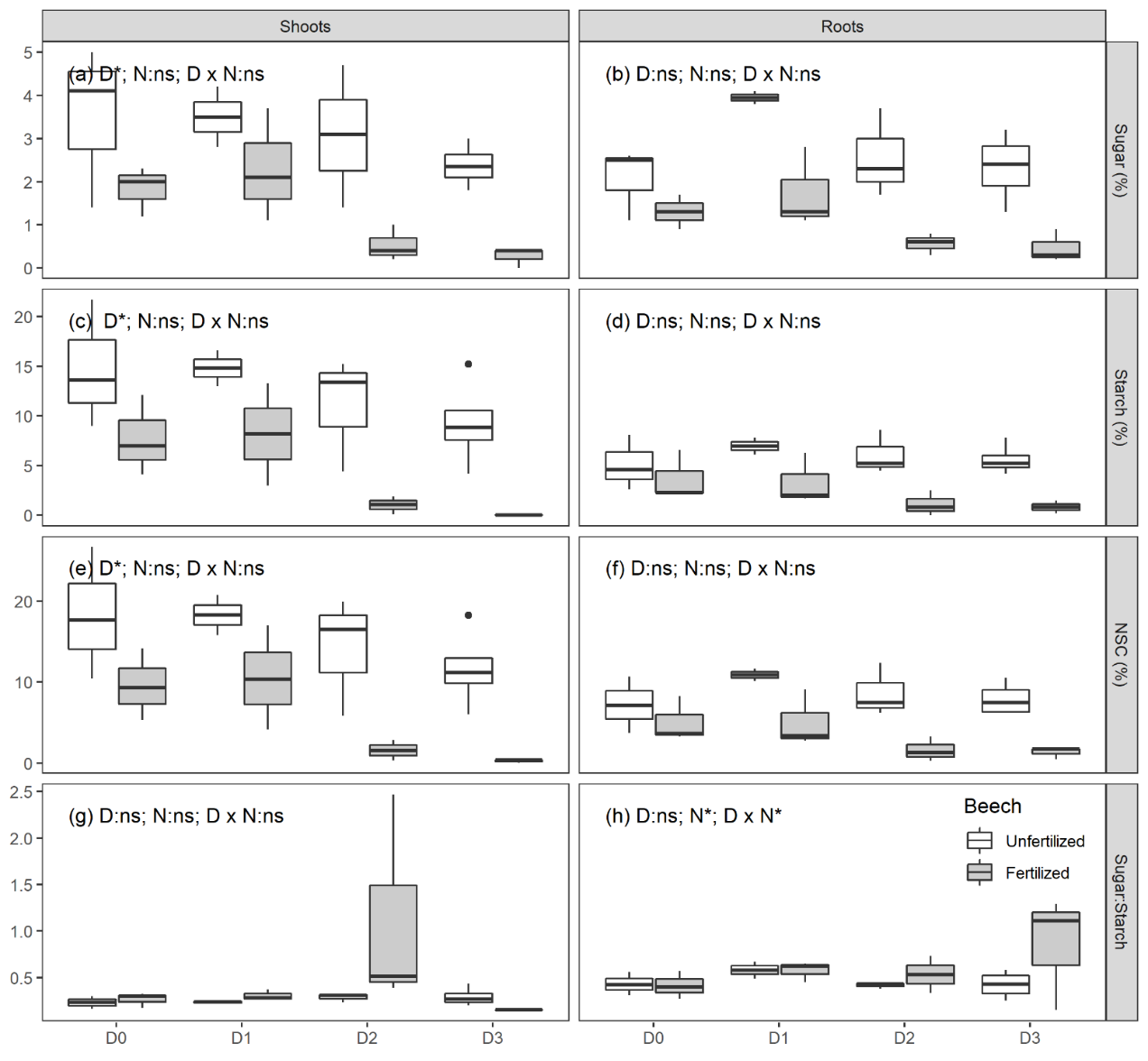


Fig. A. 7 Post-winter levels of soluble sugars, starch, and non-structural carbohydrate (NSC), as well as the ratio of sugars to starch in stems and roots of beech (*Fagus sylvatica*) after the 2018 growing season treatment with four drought durations in combination with N-fertilization.


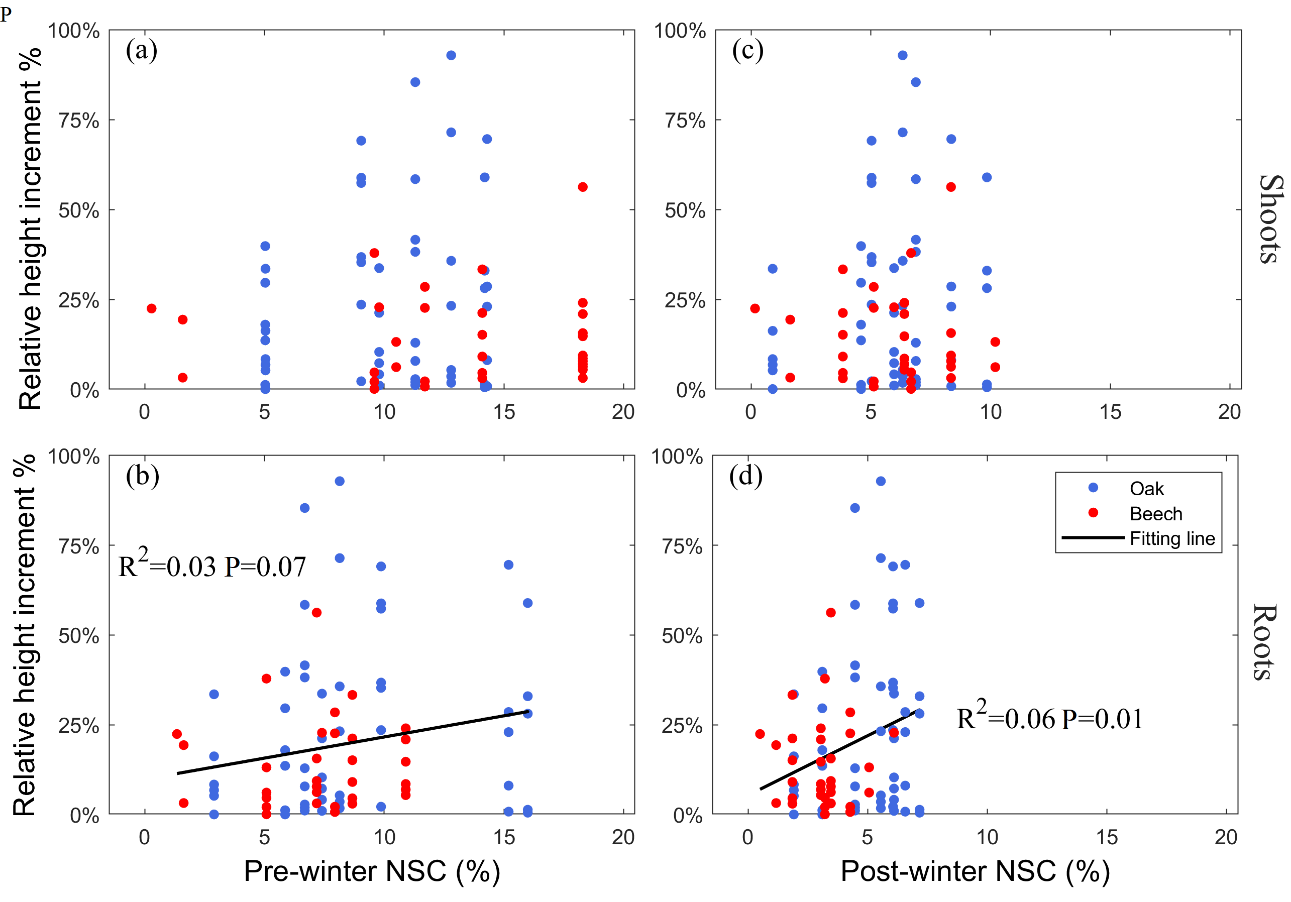


Fig. A. 8 2019 recovery growth rate (measured at the end-season 2019) in relation to pre-winter and post-winter NSC in shoots and roots of oak (*Quercus petraea*) and beech (*Fagus sylvatica*) saplings treated with four drought durations in 2018 and well-watered in 2019. In case there is a significant (p < 0.05) relationship between growth and NSC level for the pooled data, a fitting line is given.
